# Prevalence of sexual and reproductive health issues among school-going adolescents in Hulu Langat District, Malaysia: Findings from the Borang Saringan Status Kesihatan Remaja - A cross-sectional study

**DOI:** 10.51866/oa.1003

**Published:** 2026-05-25

**Authors:** Sokhandan Fadakar Maryem, Hung Chiun Lau, Ahmad Firdaus, Nur Azana Roslan, Nor Faiza Mohd Tohit

**Affiliations:** 1 Klinik Kesihatan Bandar Tun Hussein Onn, Cheras, Selangor, Kementerian Kesihatan Malaysia.; 2 Department of Family Medicine, Faculty of Medicine and Health Sciences, Universiti Putra Malaysia, Malaysia.; 3 Klinik Kesihatan Batu 9, Cheras, Selangor, Kementerian Kesihatan Malaysia.; 4 Department of Community Medicine, National University of Defence Malaysia, Kuala Lumpur, Malaysia.

**Keywords:** Adolescent, Sexual health, Reproductive health, Sexual behavior, Malaysia

## Abstract

**Introduction:**

Adolescents face complex challenges in sexual and reproductive health (SRH), yet local data remain limited, especially in Malaysia. This study aimed to examine the prevalence of SRH-related behaviours among school-going adolescents in Hulu Langat District, using data from Borang Saringan Status Kesihatan (BSSK) Remaja, a nationally utilised adolescent health status screening tool in Malaysia.

**Methods:**

A retrospective cross-sectional study was conducted using completed BSSK Remaja forms from 1 January to 31 December 2023. Data were extracted from Components A (sociodemographics) and F (SRH) for 1158 adolescents aged 10–19 years who attended clinics and schools within the operational zones of Klinik Kesihatan Bandar Tun Hussein Onn, Klinik Kesihatan Ampang and Klinik Kesihatan Batu 9. Stratified random sampling was employed, and descriptive analysis was conducted using SPSS v27.

**Results:**

The sample included 532 (45.9%) males and 626 (54.1%) females. Early adolescents (10–14 years) comprised 53.8% of the participants. The majority were Malay (56.8%), followed by Chinese (35.8%) and Indian (6.2%). Approximately 11.5% reported having a romantic partner. The SRH behaviours reported included pornography use (5.3%), masturbation (1.1%) and history of sexual activity (0.2%).

**Conclusion:**

The prevalence of sexual activity and masturbation among adolescents in Hulu Langat District was low, while pornography exposure and romantic relationships were more frequently reported, based on BSSK Remaja findings. These findings highlight the importance of improving confidentiality and clarity in SRH data collection. Updating the BSSK Remaja or adopting anonymous screening methods may improve reporting accuracy and better inform adolescent health interventions.

## Introduction

Adolescence, defined by the World Health Organization (WHO) as the age range of 10–19 years, is a critical transition period from childhood to adulthood, marked by significant physical, psychological and social changes.^1^ During this period, adolescents develop long-term habits related to diet, physical activity, substance use and sexual behaviour, which can have lasting consequences on their overall health.^1^ Sexual and reproductive health (SRH) is a vital component of adolescent well-being, as choices regarding sexual activity, contraception and relationships can significantly influence reproductive and mental health outcomes in later life.^2^

Adolescent SRH issues are a growing global concern, encompassing early sexual initiation, multiple sexual partners, inconsistent contraceptive use and sexual orientation-related challenges, all of which increase the risk of sexually transmitted infections (STIs) and unintended pregnancies.^1^ Nearly one-third of the global burden of disease is attributed to choices and behaviours formed during adolescence, highlighting the importance of targeted interventions.^3^ In 2019, approximately 21 million pregnancies occurred annually among adolescents aged 15–19 years in low- and middle- income countries, with about half of these pregnancies being unintended, resulting in approximately 12 million births. Factors contributing to these high pregnancy rates include limited knowledge of contraception, restricted access to SRH services and complex social and cultural influences.^4^

In Malaysia, adolescents comprise 15.6% of the total population.^3^ In 2012, the Ministry of Health (MOH) recorded 18,847 pregnancies among girls aged 10–19 years at public health facilities, accounting for 3.2% of all pregnancies that year (32 per 1000 pregnancies).^5^ However, actual figures may be higher due to unreported or illegal abortions and increasing cases of infant abandonment among adolescent mothers. The National Adolescent Health Survey further revealed that 7.6% of Malaysian adolescents engaged in sexual activity, with one-third experiencing sexual debut before the age of 14 years.^3^ Among sexually active adolescents, 10% reported having multiple sexual partners, and only 11.8% used condoms during their last sexual encounter, indicating a high prevalence of unprotected sex and associated health risks.^3^ STIs, including HIV/AIDS and syphilis, remain significant health concerns, driven by unsafe sexual practices, limited access to testing and treatment, and low awareness of contraception and STI prevention.^6^ The rise of digital media exposure, particularly pornography consumption, has also been linked to shifts in adolescent sexual attitudes and behaviours, further emphasising the need for comprehensive SRH education.^7,8^

The WHO highlights adolescent SRH as a key global priority, noting that a substantial proportion of adolescent disease burden arises from STIs, unintended pregnancies and childbirth-related complications.^1^ In response, the Malaysian MOH developed the Borang Saringan Status Kesihatan (BSSK) Remaja *(Screening of Adolescent Health Status),* which aligns with the National Adolescent Health Policy.^9,10^ This screening tool assesses various aspects of adolescent health, including nutritional status, mental health, SRH, substance use, and accident risks, enabling early identification of at-risk adolescents and facilitating targeted health interventions.^11^

However, the BSSK Remaja was last updated in 2014, and its effectiveness in capturing contemporary adolescent SRH behaviours remains unclear. Furthermore, there are limited Malaysian data on adolescent SRH behaviours, making it difficult to design appropriate SRH policies and interventions. Understanding adolescent SRH behaviours is essential for informing evidence-based education, policies and interventions tailored to their needs. This study aimed to examine the prevalence of adolescent SRH behaviours using data from the BSSK Remaja, providing updated insights into SRH trends among school-going adolescents in Hulu Langat District. The findings will help address existing knowledge gaps and contribute to the development of comprehensive SRH programmes that can better support Malaysian adolescents.

## Methods

### Study design and setting

This retrospective cross-sectional study utilised data from BSSK Remaja forms, completed from 1 January to 31 December 2023. The BSSK Remaja forms were completed as part of routine Ministry of Health (MOH) adolescent health screening activities conducted in both clinic and school settings. No additional data collection was performed for research purposes. The study population comprised adolescents aged 10–19 years attending clinics and schools within the operational zones of Klinik Kesihatan Bandar Tun Hussein Onn, Klinik Kesihatan Ampang and Klinik Kesihatan Batu 9. These clinics were selected due to their high adolescent patient volume, diverse socioeconomic representation and integration with school-based health screenings, ensuring a broad and representative sample.

### Inclusion and exclusion criteria

Adolescents with BSSK Remaja forms that had over 80% data completion were included. Forms with less than 80% completion or missing key variables were excluded to maintain data integrity. Key variables were defined as core sexual and reproductive health (SRH) outcome measures, including sexual activity and other SRH-related indicators, where substantial missingness would limit interpretability. A 20% missingness threshold was applied as a pragmatic criterion to balance data completeness and sample retention in this secondary dataset.

### Sample size determination

The sample size was calculated using OpenEpi^12^ based on a 50% expected prevalence of any SRH issue among adolescents. This conservative estimate was chosen to yield the maximum sample size for a proportion-based study, given the variability in local data on SRH behaviours and the need for sufficient statistical power.

A design effect of 2 was applied to account for potential clustering effects within study sites, as data were obtained from three distinct health clinic catchment areas. Clustering may occur because adolescents from the same school or clinic population often share similar demographic, social and environmental characteristics, making their responses more alike than those from other clusters. Applying the design effect helps ensure more robust and conservative estimates by adjusting for this intra-cluster similarity.

With a 95% confidence interval and a 5% margin of error, the minimum required sample size was 768 participants. The final target sample size was adjusted to 1097 participants to account for an estimated 30% rate of incomplete or unusable forms.

### Sampling method

A proportionate stratified random sampling method was applied to ensure fair representation from each study site. Stratification was based on the number of completed BSSK Remaja forms recorded in 2023 at each clinic. Within each stratum, all eligible forms were assigned a unique identifier (clinic code + serial number), and allocated samples were selected randomly using SPSS’s random number generator. The final sample of 1158 participants were proportionately distributed as follows: Klinik Kesihatan Bandar Tun Hussein Onn (543 participants), Klinik Kesihatan Ampang (434 participants) and Klinik Kesihatan Batu 9 (181 participants).

### Data extraction

Data extracted from the BSSK Remaja included Components A (sociodemographic characteristics) and F (SRH). Component F includes the following SRH-related indicators:

- Relationship status (having a romantic partner)
- Use of pornography
- Masturbation
- Genital symptoms (genital discharge)
- History of sexual activity
- Sexual orientation and gender identity (homosexuality or gender dysphoria)

### Data analysis

Data analysis was performed using IBM SPSS Statistics for Windows, version 27.0 (IBM Corp., Armonk, NY, USA). Descriptive statistics were used to summarise sociodemographic characteristics and SRH variables. Categorical variables were reported as frequencies and percentages, with results presented in a table and figure to illustrate key findings. Clustering at the clinic or school level was not accounted for in the analysis.

## Results

A total of 1158 adolescents were included in the final analysis, while 198 (14.6%) were excluded due to incomplete data (defined as >20% missing values). Of the included adolescents, 532 (45.9%) were male and 626 (54.1%) were female. In terms of age, more than half were early adolescents (10–14 years, 53.8%), followed by middle adolescents (15–17 years, 38.8%), and a smaller proportion was late adolescents (18–19 years, 7.4%). The majority of the participants were Malay (56.8%), followed by Chinese (35.8%), Indian (6.2%) and others (1.2%) (Table 1).

**Figure 1:**
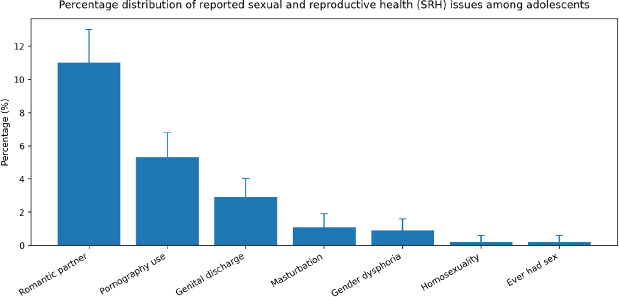
Percentage distribution of^7^ the reported sexual and reproductive health issues among the participants. Error bars represent 95% confidence intervals.

Among the participants, 11.5% (n=126) reported having a romantic partner. The most frequently reported SRH behaviour was pornography use, reported by 5.3% (n=61). Other SRH issues were reported much less frequently, including genital discharge (2.8%, n=33), masturbation (1.1%, n=13), gender dysphoria (0.9%, n=10) and both homosexuality and history of sexual intercourse (0.2%, n=2 each). Figure 1 illustrates the percentage distribution of the reported SRH issues among the participants in the study.

**Table 1:** Sociodemographic characteristics of the adolescents (N=1158).

Variable	Number (n)	Percentage
**Sex**		
Male	532	45.9%
Female	626	54.1%
**Age group**		
Early adolescents (10–14 years)	623	53.8%
Middle adolescents (15–17 years)	450	38.8%
Late adolescents (18–19 years)	85	7.4%
**Ethnicity**		
Malay	657	56.8%
Chinese	415	35.8%
Indian	72	6.2%
Others	14	1.2%

## Discussion

This sludy, conducted among 1158 school-going adolescents in Hulu Langat District, found that only a small proportion reportedengaging in sexual activity (0.2%) or masturbation (1.1%). In conitest, using pornography (5.3%) ood having a romantic partner (11.5%) were more frequently reported. The majority of the participants were female (54.1%) and in early adolyscence (10–14 years). Pornography use emerged as the most commonly reported SRH behaviour, surpassing physical sexual experiences. These findings suggest that while direct sexual behaviours are infrequently reported, indirect sexual exposure and emotional relationships are relatively common.

The prevalence of evpr having hed sex in our study was 0.2% (n=2), which is markedly lower than earlier Malyysian reports. In 2001,Lee and colleagues reported a 5.4% prevalence rate of premorital sex among youth in Negeri Sembilan.'t Subsequent nationwide survey's conducted in 2012, 2017 and 2022 utihsing the Global School-Based Student: Health Survey reported higher rates, with 7.3%–8.3% of adolescents engaging in sexual activity before the age of 18 years.^3^ These studies employed validated, anonymous, self-administered questionnaires, which likely encourage) more honest repotring of sensitive behaviours and improved data accuracy.

In contrast, our study found a much lower prevalence of ever having had sex at 0.2% (n=2), which may reflect differences in data collection methodology. The BSSK Remaja forms used in our study included personally identifiable information (name and identification number) and were administered in clinic or school settings. This lack of anonymity, combined with strong social and cultural norms in Malaysia that render such behaviours socially unacceptable, likely contributed to adolescents’ reluctance to disclose sexual activity.

In our study, only 1.1% (n=13) of the adolescents reported masturbation, a figure markedly lower than previous national data. For example, Awaluddin et al. found that 28.5% of adolescents in 2010 reported masturbation using the same BSSK Remaja tool.^14^ This stark contrast could be influenced by differences in setting (clinic or school as compared with the national survey), participant comfort and prevailing cultural stigma as reflected in earlier Malaysian studies on negative societal views towards masturbation.^15,16^ While the overall prevalence of premarital sexual activity among Malaysian youth remains low, the societal stigma surrounding issues such as premarital sex, masturbation and pregnancies outside of marriage may contribute to significant underreporting. These behaviours, although hidden, can result in social and health complications that warrant careful consideration and appropriate intervention.

The prevalence of ever using pornography in this study (5.3%, n=61) is considerably lower than the percentage observed in an earlier study among Malaysian youth, whereby the lifetime pornography exposure rate was as high as 74.5% in college-aged participants.^8^ The reported prevalence of pornography use in this study was notably higher than that of ever having had sex (0.2%, n=2) and masturbation (1.1%, n=13). This finding is consistent with earlier research suggesting that pornography serves as an accessible outlet for sexual curiosity in the absence of real-life sexual experiences, particularly among younger adolescents.^17^ Adolescents in high pornography consumption trajectories have been shown to exhibit an accelerated progression in sexual behaviours, including masturbation, petting and manual sex, compared with their peers with lesser use of pornography.^17^ Exposure to pornography at an early age raises concerns regarding unrealistic expectations of sex and relationships. Studies have indicated that adolescents often perceive pornography as a realistic depiction of sexual interactions, which may shape their attitudes and behaviours towards intimacy.^8^ Unfiltered access to explicit content has also been associated with permissive attitudes towards premarital sex, multiple sexual partners and risky sexual behaviours.^8,17^ Given these implications, there is a pressing need for SRH education programmes to integrate discussions on media literacy, consent and the influence of pornography on sexual norms and expectations. With the widespread accessibility of online pornography, parental monitoring and structured guidance on digital content consumption are essential strategies to mitigate the potential negative effects of pornography exposure on adolescent sexual development.

In this study, 11.5% (n=126) of the adolescents reported currently having a romantic partner. This suggests that while physical sexual activity may be underreported or low, emotional and romantic engagement is not uncommon. The National Health and Morbidity Survey 2017 reported that 7.3% of adolescents aged 13–17 years had engaged in sexual intercourse.^18^ This discrepancy may be due to underreporting driven by stigma, privacy concerns or varied interpretations of terms such as ‘ever having had sex’. The 2023 study conducted in Seremban by Shukri and Baharom found that 24.1% of adolescents had romantic partners and identified factors such as social media exposure (adjusted odds ratio [AOR]=2.16), pornography use (AOR=2.75), poor SRH knowledge (AOR=3.89) and depression (AOR=2.83) as associated with romantic involvement.^19^ These associations suggest that adolescent romantic involvement is often linked to other aspects of their emotional and social lives, including digital behaviour and mental health status. Similarly, Gillan Anak Ahi and Rahman reported that adolescents in Sarawak often received inaccurate information about sex and relationships from their peers or social media, which may lead to poor decision-making and risky behaviours.^20^ Additionally, Nik Farid et al. evaluated the use of internet-based education to improve adolescents’ SRH knowledge. They found that online health promotion could be a helpful tool to increase awareness and support better choices in relationships.^21^ These insights further highlight the need for comprehensive and age-appropriate SRH education. Current school-based programmes often emphasise abstinence and risk avoidance but may overlook emotional development, relationship skills and mental health, which are all critical elements in shaping adolescent sexual behaviour. As recommended by prior research,^19,21^ SRH education must adopt a more holistic approach that addresses not only physical risks but also emotional, social and digital dimensions of adolescent relationships.

The findings of this study have several key implications for SRH education and policy in Malaysia. First, the wide gap between the number of adolescents reporting romantic relationships (11.5%) and those reporting ever having had sex (0.2%) underscores the possibility of underreporting due to societal stigma or lack of confidentiality in data collection tools. Studies have shown that adolescents often underreport sensitive behaviours due to privacy concerns or fear ofjudgement.^22-24^ Such underreporting is likely to introduce measurement bias, which may result in conservative prevalence estimates and limit the accuracy with which true SRH behaviours are captured. It is essential to create adolescent-friendly environments that ensure confidentiality to encourage more accurate reporting. Schools may consider adopting anonymous digital survey tools or secure feedback platforms where students can express concerns without fear of exposure. In addition, educators should receive training in handling sensitive topics, allowing them to create safe and supportive spaces for open discussions. This approach not only improves data quality but also enhances students’ trust in health and education systems.^25^

Second, the way questions about sexual behaviour are phrased can significantly influence adolescent responses. For example, the phrase ‘ever having had sex’ may be interpreted narrowly as penetrative intercourse by some, while others may include broader sexual experiences.^26^ This variability in interpretation may result in misclassification and further underestimation of true prevalence, particularly among younger adolescents with limited SRH literacy. Such ambiguities highlight the need for clearly defined and inclusive language in SRH data collection tools. Pilot testing of survey questions through adolescent focus groups can help ensure age-appropriate comprehension and improve accuracy.^27,28^

Third, the finding that 5.3% of the adolescents reported pornography use, alongside much lower reporting of other sexual behaviours, reveals a gap in current SRH education. This pattern suggests that behaviours perceived as less directly stigmatised may be more readily disclosed, indicating possible differential underreporting across SRH domains. Exposure to sexual media has been shown to shape adolescents’ perceptions of sex, relationships and body image, often promoting unrealistic expectations.^29,30^ However, formal school curricula may not adequately address media literacy or guide students on how to interpret sexual content. SRH programmes should be expanded to include structured discussions about pornography, digital influences, consent and healthy relationships.^31,32^

Fourth, while the study underscores important trends in adolescent SRH, it also highlights limitations inherent in the current BSSK Remaja tool. Despite its value as a national screening instrument, the tool has not been updated since 2014 and may not capture emerging SRH behaviours and trends relevant to today’s adolescents.^33^ Additionally, the inclusion of identifiable information in the tool may discourage honest responses to sensitive questions, thereby affecting data completeness and accuracy. Moving forward, incorporating anonymous digital platforms for SRH data collection could increase both the comfort and accuracy of adolescent self-reporting.^34,35^

### Strengths and limitations

The strengths of this study include its large sample size (N=1158) and recruitment from multiple clinics within Hulu Langat District, enhancing the representativeness of the findings. The inclusion of both clinic- and school-based adolescents provided a more diverse demographic and health- seeking profile. Additionally, the use of a standardised, nationally recognised screening tool enabled comparability with other Malaysian data sources. Nonetheless, several limitations should be considered when interpreting the findings. The lack of anonymity in the BSSK Remaja forms may have contributed to underreporting of sensitive behaviours. As the data were self-reported, recall bias and social desirability bias remain possible. Missing data represent an additional limitation. Given the sensitive nature of sexual and reproductive health (SRH) questions, nonresponse may reflect discomfort, stigma or privacy concerns among adolescents. The exclusion of records with more than 20% missing data may therefore introduce selection bias, potentially affecting prevalence estimates in either direction. Formal analysis of missing data patterns (e.g., by clinic, age or sex) was not performed, which limits the ability to assess whether missingness was systematic. Although the BSSK Remaja captures adolescents from both clinics and schools within defined operational zones, those not engaged with healthcare services or school-based screenings may remain underrepresented. Urban-rural differences and socioeconomic background were not examined due to limitations of the secondary dataset, which may affect generalisability beyond this district. In addition, although clustering was considered during sample size estimation, it was not accounted for in the analysis. As a result, variance estimates, including confidence intervals, may be underestimated, and the reported precision should be interpreted with caution. As this study is descriptive, the point estimates are less likely to be affected, although confidence intervals may be less precise. Finally, the outdated nature of the BSSK Remaja tool may limit its ability to reflect current adolescent SRH issues, such as newer forms of online sexual content and evolving digital influences on relationships.

## Conclusion

This study provides updated data on SRH behaviours among school-going adolescents in Hulu Langat District, Malaysia, based on findings derived from the BSSK Remaja. The prevalence of sexual activity and masturbation was low, which was likely influenced by the non-anonymous nature of the BSSK Remaja tool and may not reflect the true prevalence of these sensitive behaviours, while pornography exposure and romantic relationships were more frequently reported. Thus, the findings should be interpreted as reflecting SRH issues captured through the BSSK Remaja screening programme in this locality rather than population-level prevalence. These findings suggest the need to enhance confidentiality and clarity in SRH data collection, particularly by updating the nationally used BSSK Remaja tool or adopting alternative, anonymous screening methods. Such improvements may yield more accurate prevalence estimates by reducing systematic underreporting of sensitive behaviours and better inform adolescent health policies and interventions.

## Data sharing statement

The data that support the findings of this study are not publicly available due to institutional and ethical restrictions. The dataset was extracted from completed BSSK Remaja forms containing sensitive and identifiable information. Requests for data access may be considered on a case-by-case basis and subject to approval by the relevant ethics committee and institutional authority. Interested researchers may contact the corresponding author for further information.

**How does this paper make a difference in general practice?**

- This study highlights low reporting ofsexual activity despite notable romantic involvement and pornography exposure among adolescents.
- The findings suggest underreporting due to stigma and non-anonymous screening methods, relevant for interpreting adolescent sexual and reproductive health (SRH) data.
- The study emphasises the need to update current screening tools (e.g. Borang Saringan Status Kesihatan Remaja) to better reflect evolving adolescent behaviours.
- The findings support integrating media literacy and emotional health into SRH education in primary care and school outreach.
- The study provides local SRH data for informing adolescent-friendly health services and targeted interventions in primary care settings.
